# Development of a childhood obesity prevention programme with a focus on UK South Asian communities^☆^

**DOI:** 10.1016/j.ypmed.2013.08.025

**Published:** 2013-12

**Authors:** Miranda Pallan, Jayne Parry, K.K. Cheng, Peymané Adab

**Affiliations:** a School of Health and Population Sciences, University of Birmingham, Edgbaston, Birmingham B15 2TT, UK

**Keywords:** UK, South Asian, Child, Obesity, Prevention

## Abstract

**Objective:**

We report the development of a childhood obesity prevention intervention for UK South Asian primary school-aged children, guided by the UK Medical Research Council (MRC) framework for complex intervention development and evaluation.

**Methods:**

We combined information gained from a literature review, stakeholder focus groups, an expert group, review of local resources and mapping to the Analysis Grid for Environments Linked to Obesity (ANGELO framework) in an intervention development process. The study took place in 2007 in Birmingham, UK.

**Results:**

Contextual information from the stakeholder focus groups was essential for informing intervention development. The expert group defined guiding principles for the intervention. Informing intervention design by assessing existing local resources addressed intervention sustainability. The use of the ANGELO framework ensured a comprehensive environmental approach to intervention development. The intervention consisted of two broad processes; increasing children's physical activity levels through school, and increasing skills of families through activity-based learning. The developed intervention is being evaluated in a major study.

**Conclusions:**

The intervention development process has resulted in a tailored intervention programme to prevent childhood obesity in UK South Asian communities, but also intervention processes that could be applied to other communities and tailored to local context.

## Highlights

• We describe the process of development of a childhood obesity prevention programme.
• We combined findings from multiple methods in an iterative process of development.
• Childhood obesity prevention interventions need to be tailored to the local context.
• Understanding the local context is critical for intervention tailoring.

## Introduction

Childhood obesity is a global threat to health (World Health Organization, 2000). Much obesity prevention research has been undertaken in the last two decades but the “key ingredients” of successful programmes remain unclear (Brown and Summerbell, 2009; Doak et al., 2006; Flodmark et al., 2006; Waters et al., 2011). In part, this may reflect the critical roles which population-specific social norms and context play in mediating an intervention's effectiveness and which thus must be accounted for when developing new preventive strategies (Summerbell et al., 2005).

Understanding context is particularly important when developing interventions for specific cultural communities, as shown by childhood obesity prevention studies targeting minority ethnic groups in the USA (American Indian children; Gittelsohn et al., 1999) and the UK (South Asians; Pallan et al., 2012). For example, in the latter, there is much concern around children being underweight, especially among older community members, and hierarchical family structures result in grandparents exerting control over children's lifestyle behaviours. Understanding these norms and beliefs forms a critical foundation on which the intervention development process can begin.

UK South Asian children are a specific target group for obesity prevention as they are particularly vulnerable to the short and long-term health consequences of obesity (Bhopal et al., 1999; Whincup et al., 2002). In this paper we describe the development process of a childhood obesity prevention intervention targeting primary school-aged children from this cultural group (the UK National Prevention Research Initiative-funded BEACHeS study). Specifically we reflect on the utility of a well-recognised complex intervention development framework tool (the MRC Framework; Campbell et al., 2000) as a means to ensure that contextual information is gathered and incorporated into the intervention design. This is analogous to stage 1 of the NIH Stage Model (Onken et al., 1997), which emphasises the importance of incorporating qualitative research methods into intervention development.

## Methods

The stages outlined in the MRC Framework (Campbell et al., 2000) and also in the Stage Model (Onken et al., 1997) are akin to the sequential phases of drug development. The theoretical phase (preclinical/Stage 0) and modelling phase (phase I/Stage 1a) inform the development of behavioural interventions prior to feasibility or exploratory testing (phase II/Stage 1b), and precede the more definitive clinical trial and implementation phases (phases III–IV/Stages 2–5). In this study, the methodologies employed were a literature review on childhood obesity prevention, focus groups (FGs) with local stakeholders, a Professionals Group meeting, and a review of existing community resources. Each of these is discussed in turn below.

A further theoretical framework was used to assist in the analysis and application of the contextual data during the intervention development process; the Analysis Grid for Environments Linked to Obesity (ANGELO framework; Swinburn et al., 1999). This framework guides users to categorise ‘obesogenic’ environmental influences into four types: physical, economic, political and sociocultural, and consider these categories at both local and macro-levels. Data arising from the literature review and the stakeholder FGs were mapped to this framework, which was then used to inform decisions on components to include in the final intervention programme.

### Literature review

We systematically searched the Cochrane, MEDLINE and the NIHR Centre for Reviews and Dissemination databases for childhood obesity prevention systematic reviews and evidence-based guidelines to ensure that the developed intervention was coherent with the existing evidence. In addition, the following websites were searched: National Institute for Health and Clinical Excellence, NIHR Health Technology Assessment Programme, Scottish Intercollegiate Guidelines Network, and Swedish Council on Health Technology Assessment. Publications up to the end of 2006 were included in the review.

We dissected intervention programmes reported in the literature into their component parts. These were summarised into meaningful groups, for example, ‘activities for families’ or ‘increased extracurricular physical activity provision in school’. This information was presented in the stakeholder FG sessions to facilitate discussion on the most effective and feasible types of intervention for their local communities.

### Focus groups with stakeholders

We recruited adult stakeholders from eight school communities in Birmingham, UK to participate in FGs. A detailed description of recruitment and FG procedures is described elsewhere (Pallan et al., 2012). Stakeholders included parents, teachers, school catering staff, other school support staff, school governors, healthcare professionals, local authority representatives, religious leaders, leisure centre staff, and retail representatives. Nine FGs were convened comprising 68 participants (88% female; 55% South Asian). Each group met for two sessions (70% attended both sessions). The aim of the FGs was to reach consensus on up to eight intervention components that participants believed would warrant inclusion in an intervention programme for their local communities, given the perceived importance and feasibility of implementation.

FGs were audio-recorded and transcribed. Analysis was two-staged. First an inductive thematic analysis was undertaken to identify themes relating to conceptual influences on the development of childhood obesity (findings described elsewhere; Pallan et al., 2012). Second, data on ideas for childhood obesity prevention, barriers and facilitators to intervention, and the balance given to importance and feasibility of each component were extracted from the transcripts (data presented in this paper). To assist with this process a framework for data extraction was developed prior to analysis. This second analysis was a more deductive process, recognising that this is an appropriate approach when undertaking applied qualitative research that has preset aims and objectives (Pope et al., 2000).

### Review of local resources

A systematic approach to mapping local community assets was developed, which included discussion with school, health and local community representatives, internet searches and visits to the communities. The purpose was to enable the intervention programme to build on existing resources, thus making it more relevant to local communities and more sustainable.

### Professionals group

A Professionals Group was established to advise on intervention development. The Group consisted of nutritional, physical activity and behavioural epidemiologists, health psychologists, a dietician, an obesity programme commissioner, a paediatrician, a qualitative researcher, an educationalist and experts in ethnic minorities research. The role of the Group was to consider the FG data and the existing literature, and to advise on components to be included in the final programme.

## Results

### Literature review

Eight relevant systematic reviews were identified (Bautista-Castano et al., 2004; Doak et al., 2006; Flodmark et al., 2006; Hardeman et al., 2000; NHS Centre for Reviews and Dissemination, 2002; Sharma, 2006; Stice et al., 2006; Summerbell et al., 2005), encompassing 70 studies. Despite study heterogeneity, findings from the eight reviews were relatively consistent: most interventions showed some positive effect on obesity-related behaviours (food intake, physical activity or sedentary behaviours), but the effects on outcome measures of overweight and obesity were at best modest. Potentially effective intervention strategies highlighted by reviews included targeting sedentary behaviours (Bautista-Castano et al., 2004; Doak et al., 2006; NHS Centre for Reviews and Dissemination, 2002; Sharma, 2006), involving parents, and longer intervention duration (Bautista-Castano et al., 2004). From among the included studies, 23 intervention components were identified and classified according to the setting for delivery, and the constituent activity (Table 1).

### Findings from stakeholder focus groups

Several intervention themes emerged from the FGs. The importance of targeting parents and families was highlighted by all groups. Most participants recognised that schools are a facilitator to intervention in that they provide a gateway to parents (especially mothers), and so provide a channel through which family interventions can be delivered. Accessing fathers and extended family members was also acknowledged to be important but deemed difficult to achieve. Educational activities for families and interventions to increase parenting skills emerged as priorities for several groups. There was emphasis on educational interventions aiming to confer skills, rather than knowledge. Written educational materials were felt to be largely ineffective in the target population because of low literacy levels.

School-based interventions were extensively discussed and it was recognised that there was much ongoing activity related to healthy behaviours, partly linked to UK national directives (Department for Education, 2012; School Food Trust, 2012). Participants felt that coherence of new initiatives with other demands on the school, for example the delivery of the national curriculum, would be facilitatory. Increasing physical activity in the school day outside of the physical education curriculum was widely perceived to be important and feasible. Provision of out of school physical activities was also felt to be important and was frequently included in groups' final priority lists. Accessibility to these activities in terms of location, timing, cost, and cultural acceptability and interests was perceived as important. Particular cultural barriers to out of school physical activities were highlighted, including low acceptability of sportswear for Muslim women, and the daily requirement of attending mosque after school for Muslim children.

Improving the nutritional value of school meals and access to healthy foods in school was frequently discussed. Some participants felt that school nutrition was very important, but others felt that food intake out of school was more important to address. Professional participants noted that existing national policy was already addressing nutrition in schools and saw this as a facilitator to further nutrition intervention beyond schools.

Rewards for healthy behaviours was widely perceived to be a feasible intervention, but of doubtful effectiveness. Involvement of children in the planning of delivery of interventions through mechanisms such as school councils was felt to be feasible and an important facilitator to intervention.

The importance of adult role models in influencing healthy behaviour was a repeated theme. Potential role models included parents, teachers, celebrities, and faith leaders. The central role of religion in the predominantly Muslim communities was seen as an opportunity for intervention, therefore faith leaders were a particular focus of discussion. Several participants (but not all) felt that faith leaders would not engage with interventions targeting health-related behaviours as they would not identify this as part of their ‘faith’ role.

There was a general perception that the community setting provided an unexploited opportunity. The provision of local low cost physical activity and healthy eating sessions were suggested and prioritised as potential interventions. Existing community resources were identified as a potential opportunity and effective signposting to these was a suggested intervention. Quotations from participants illustrating emergent themes are shown in Table 2.

In addition to the specific facilitators and barriers discussed, several global barriers to intervention emerged. Children's expectations and demand for choice was a perceived barrier; children expect to have a choice of food at home and school, and prefer sedentary activities such as television and computers. Cultural norms within South Asian communities were highlighted. Practical barriers such as language were perceived to make intervention more challenging, but a more fundamental barrier identified was the perception that overweight children are healthy, and underweight is of more concern. The lack of parental time was identified as a barrier (see Discussion). This would impact on any interventions involving parental engagement. A further perceived barrier was the cost of commodities such as healthy food and leisure time activities. The major barriers identified for schools were curricular pressures and competing priorities. Quotations from participants relating to intervention barriers are shown in Table 3.

### Professionals group

After consideration of the FG data, the Professionals defined a set of underlying principles to guide intervention design and delivery. These were: development of an inclusive (suitable for all ethnic groups), sustainable intervention; a focus on developing practical skills; delivery of the intervention in a predominantly verbal format; and involvement of children in the implementation planning. The group then agreed a shortlist of components to be considered for the final programme. These included: family educational activities to confer practical skills; increasing provision of physical activity within the school day; provision of lunchtime and after school activities; and community-based walking interventions. The group felt that rewards could be linked to some of these components. Although intervention in faith settings such as mosques would access children from Islamic families, the Group was concerned that this would exclude non-Islamic families and therefore would not fit with the principle of inclusivity.

### Review of local resources

The local resource review revealed many ongoing initiatives implemented by the health, education, and voluntary organisations. Examples include food skills courses for parents, provision of school gym equipment, a dietician working with schools, healthy eating and physical activity courses at a local Premier League Soccer Club, active travel to school plans, structured play resources for schools, community walk leader schemes, and a variety of sports and physical activity clubs and facilities.

### Mapping to the ANGELO framework

The intervention activities identified from the literature (Table 1) spread across all four environment types. Interventions prioritised by stakeholders however, addressed the physical, political and sociocultural more frequently than the economic environment. In the final intervention programme, all environment types are addressed, with the greatest emphasis on the physical environment (Table 4).

### The intervention development process

**Fig. 1:**
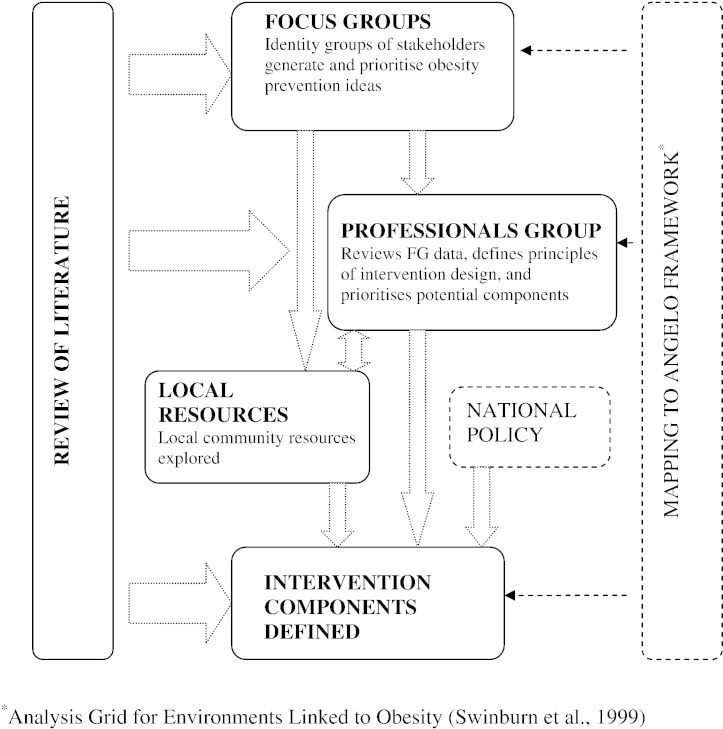
Processes undertaken to develop a childhood obesity prevention intervention for UK South Asian communities (setting: Birmingham UK, 2007).

Several important factors were identified that needed consideration within the development process. First, we recognised that the contextual information from the FGs was of key importance (described in detail elsewhere; Pallan et al., 2012). The Professionals Group had a central role in defining a set of guiding principles, and the resource review addressed the need for intervention sustainability. The study was undertaken at a time of great political focus on childhood obesity, and national policy around healthy behaviours was taken into account in the development process to ensure that the final intervention programme would be beneficial over and above ongoing national initiatives. The iterative development process is schematically represented in Fig. 1.

The final intervention programme consisted of two broad processes; increasing children's physical activity levels through school, and increasing skills of parents and families through activity based learning. The intervention components are described in Table 4.

## Discussion

This paper presents the development of a childhood obesity prevention intervention, guided by the MRC Framework (Campbell et al., 2000). Since the study started, the MRC have updated their guidance (Craig et al., 2008), bringing to the fore the need for even greater attention to early phase development work. This updated guidance recognises the importance of understanding local contexts, the need for an iterative approach and a greater emphasis on developing a prospective theoretical understanding of how the intervention will achieve the desired outcome. Our experience supports the guidance on obtaining detailed contextual information to inform development and delivery of an intervention. We gained rich data on local context from the stakeholder FGs, particularly relating to the cultural and religious practices of the communities within the study population, which shaped the intervention design.

The importance of understanding the cultural and religious context in minority ethnic communities has been highlighted in other studies. In a childhood obesity prevention study targeting minority ethnic communities in London, Rawlins reported child and parent perceptions of healthy eating and physical activity. The findings relating to South Asian communities resonate strongly with our data, for example the influence of places of worship and the role of extended family members on healthy lifestyles (Rawlins et al., 2013). A recent comprehensive evidence synthesis review on adapting health promotion programmes (including diet and physical activity) for minority ethnic groups also draws attention to the importance of tailoring to particular contexts. The authors concluded that such adaptation increased intervention relevance and acceptability, although whether this results in increased effectiveness is undetermined (Liu et al., 2012).

The need for considering local context brings up the question of intervention transferability to different settings. Hawe and colleagues argue that a complex intervention can be standardised and transferable if it is the function and process of the intervention (e.g. mechanisms to increase children's physical activity in school) that are standardised rather than the components (e.g. a specific curricular activity). This enables the delivery of interventions to take into account local context (Hawe et al., 2004). This approach necessitates a theoretical understanding of the change mechanisms of local context at each intervention site. We would argue that this is a viable approach. An understanding of the contextual factors is essential for tailoring intervention components and thus determining their success. For example, barriers to childhood obesity prevention interventions, such as lack of parental time repeatedly emerge in the literature (O'Dea, 2003; Pocock et al., 2010; Power et al., 2010; Sonneville et al., 2009). However, this barrier can only be addressed if the precise nature of the constraints on parental time is understood. In this study mothers were likely to be constrained through obligations such as looking after extended families or attendance at places of worship (Pallan et al., 2012), whereas in a North American study of white middle class children, perceived time constraints related to parents' work commitments (Power et al., 2010). Different approaches to intervention would be required to overcome this barrier in these two communities.

### Study strengths and limitations

The iterative development process enabled us to implicitly gain a theoretical understanding of change pathways, and use this to drive intervention development. This is consistent with the use of a ‘Theories of Change’ approach where researchers work with participants to surface the mechanisms by which an intervention may or may not bring about change (Weiss, 1998). In turn this permits evaluation of the implemented intervention/s to be better informed by the use of theory-driven approaches (Connell and Kubisch, 1998; Pawson and Tilley, 2009).

The validity of considering intervention components separately (as was done in the FG discussions) could be challenged, given that the effects of a complex intervention may be greater than the sum of its parts. However, the exploratory and prioritisation processes that the participants were guided through enabled them implicitly to consider individual components and the synergies between them in their local contexts. This further contributed to the development of a theoretical understanding of the change pathways interventions were likely to invoke.

Researchers may argue that the prioritised intervention components ultimately included in the intervention programme could have varied depending on factors such as the mix of FG participants or the professionals recruited. This is a frequent challenge to those working with qualitative techniques. However our analysis showed thematic concordance across groups and given our breadth of sampling we believe the prioritised outcomes are transferable within comparable communities. The information on local context gained from the groups, together with the existing resource review, was crucial in the detailed planning of programme components.

### Conclusions

The processes undertaken have led to the development of an intervention founded within existing research evidence, but also taking into account the local context. The intervention development balanced pragmatism with theory driven approaches. The result is a childhood obesity prevention programme that is tailored to UK South Asian communities, but one which could be transferred and tailored to other settings. Emergent data from similar intervention development research that we have undertaken in Iran, Qatar and China supports this approach (Al-Muraiki, 2012; Li, 2013; Mohammadpour-Ahranjani, 2011). Data gained from stakeholders in these settings has shown that the contexts that contribute to the development of childhood obesity are broadly similar, suggesting that prevention programmes could be transferred from one setting to another. However, this research has also highlighted that there are specific contextual differences that are critical to identify and understand in order to successfully tailor obesity prevention programmes to the different settings.

## Conflict of interest statement

The authors have no competing interests to declare.

## Figures and Tables

**Table 1:** Classification of intervention processes for childhood obesity intervention.

Setting	Broad description of intervention process	Type of environment targeted^a^
Family	Activities for family	Physical/sociocultural
Educational materials aimed at families	Physical/sociocultural
Motivational interventions and incentives to promote healthy behaviour in family	Economic/sociocultural
Campaigns aimed at families	Political
Develop parenting skills	Sociocultural
School: Curricular interventions	Alter physical education provision	Physical/political
Increase physical activity provision	Physical/political
Increase nutrition education	Political
Increase general health education	Political
Work on self-esteem	Sociocultural
Motivational interventions to influence behaviour change	Economic/sociocultural
School: Extra-curricular interventions	Media promotion of healthy diet and physical activity	Political
Lunchtime/after school/holiday clubs	Physical
Encourage healthy travel to school	Physical/sociocultural
Competitions/rewards to promote healthy behaviours	Economic
School: Food provision	Increase nutritional value of school meals	Physical
Improve provision of and access to healthy food in school	Physical
School: Other	School action plans/policies related to health	Political
Professional development for teachers	Political/sociocultural
Involve children in school changes	Political
Adult role models/school ‘champions’	Sociocultural
Community	Provide sessions for all community members	Physical
Work with local shops	Political/economic

a As defined by the Analysis Grid for Environments Linked to Obesity (Swinburn et al., 1999).

**Table 2:** Quotations to illustrate themes relating to childhood obesity prevention from stakeholder focus groups (setting: Birmingham UK, 2007).

Intervention themes	Stakeholder quotations
Targeting families	“*It comes down to the parents doesn't it? …whatever the school does … whether it's fruit or break snacks or lunchtime, I think it's excellent. But after that when I take them home, you know, the school doesn't know what's been put down there*” (parent: female Pakistani) “*you could offer classes in healthy eating and such like, or do keep fit and also, or something with parents, but you only get a very small amount that are coming in and you are putting in a lot of time and resource into educating a few, it's getting them in, getting at them from other channels rather than just through school*” (teacher: female white European)
Parental educational activities to confer skills	“*It's educating the parents, I think that's vital. And not in the form of just giving them notes, because most of them won't be able to read or write, I think it's going to the community, going to schools, somebody who speaks the language, interpretations of different lifestyles and different types of things, I think that would help a lot*” (parent: female Yemeni) “*if you had a parents day, where they actually brought their parents in with them, and they had like a cooking class, or classes done about healthy eating, where the parents were there with the children, learning the same things that the children are learning about food*” (parent: female Pakistani)
Increasing physical activity in the school day	“*one thing you can look at is, if they're at school and they've just had this lunch, you've got like this hour, in theory, where you've got them, someone being paid to supervise them, it's all organised. So the lunch hour and the break hour it would be maybe to look at introducing games … to get the kids out of the dinner halls, get them outside, then get them playing.*” (retail representative: male white European) “*It has to be effortless, well planned, popular, sustainable, possible to embed in school practice and school life, not require too much staff time, not rely on volunteers*” (leisure representative: male white European)
After school activities	“*from a Muslim background like myself, I can't take my children swimming because I just don't feel comfortable*” (parent: female Bangladeshi) “*When you say after school activities, we have after school clubs in school, but children don't want to go because they know they've got mosque and they haven't got time to go to activities*” (parent/school support staff: female Indian)
School meal provision	“*I totally and absolutely agree that it [nutritional content of school meals] is important, but is it, is it good enough as it is and are we just not talking about what they get at lunchtime, we are talking about what they get before and after school. So of course it is important but I just feel that it is happening already*” (community representative: female white European)
Rewards as incentives	“*Rewards for healthy lunch boxes, that is something that is really easy to do, we find a nice sticker, you do not have to do anything else, I've got healthy lunch box stickers and they work a treat*” (school-community liaison representative: female white European)
Involving children in intervention planning	“*that is why involving children in the changes [is important], you know hopefully you can encourage them to decide not to go and have their ice cream after school or whatever.*” (health representative: female white European)
Role models	“*I know that a sporting hero, you know, if someone came along, it doesn't matter who … and said you have to eat more beans, they would do it*” (teacher: female white European) “*there are so many people that go to the mosques and gurudwaras and temples and what have you, and that is the way to access all these people and the children, so working with the faith groups would be important I think.*” (community leader: female Indian) “*The most unfortunate thing is like Bangladeshi and Pakistani, a lot of mosques the Imams, they are not British, you know trained in this country. They just came from the villages, the majority of them*” (Islamic faith leader: male Bangladeshi)
Community initiatives and facilities	“*if there were people that were running community activities that could gather a group of boys in the park, and say, come on let's do a free activity*” (parent: female Pakistani) “*showing families the parks we have, you know, canal walks and things like that.*” (leisure representative: male white European)

**Table 3:** Quotations to illustrate general barriers to childhood obesity prevention intervention from stakeholder focus groups (setting: Birmingham UK, 2007).

Identified barrier	Quotation
Children's expectations and choices	“*I was at a school the other day and they were saying that they serve pizza once a week and that's the day that all the kids turn up with their lunch money …and they come with their packed lunches on other days …kids aren't stupid, they want to eat the pizza*” (retail representative: male white European)
Cultural attitudes to children's weight	“*and you get criticised as well if your children are thin. I mean, I get it all the time*” (parent: female Pakistani)
Lack of parental time	“*It's lack of time as well, the more kids you've got you have to take time out for everything because your life is so busy, you've got other children as well and you have to concentrate everywhere, you've got the household to do as well, and it's just too much*” (parent: female Pakistani)
Cost of food/leisure facilities	“*some parents might not be able to afford to pay [for sports clubs], if they have got, say, three children, and they can't pay for three because they can only pay for the one*” (school catering staff: female white European) “*Buying a massive sack of chips is what, like two pounds? And going out and buying something healthy would actually cost you far more.*” (Retail representative: male white European)
Competing priorities in schools	“*the problem is that the obvious place for putting education in place is within the school, but you are very limited … it's not our job to educate the parents*” (teacher: female white European) “*the teachers you know, they have got other things to do in the evenings and in terms of preparation and marking, and they don't want to be running all these clubs*” (health representative: female white European)

**Table 4:** Intervention components included in the BEACHeS intervention programme (setting: Birmingham UK, 2007).

Intervention component		Aim	Description	Environment addressed^a^
Increasing children's physical activity through school	Physical activities within the school day	To increase the overall amount of time that children are physically active within the school day	Three elements introduced into schools: 1. ‘Wake Up Shake Up’: a short organised daily dance or exercise routine to music 2. Organised playground activities at lunch times through the training of play leaders 3. ‘Take 10’: teaching resource which links 10 min physical activity to curricular subjects.	Physical Political
Incentive scheme to encourage physical activity out of school	To increase the amount of time outside of school hours that children spend doing leisure activities with a physical element	Children receive a sticker collection card from school and information on local participating sports and leisure venues. Each time a child attends a venue, they collect a sticker. The child with the most stickers in each school receives a prize.	Economic
Attendance at a course run by a Premier League Football Club	To encourage physical activity and a healthy diet by delivering positive health-related messages through an iconic sporting institution	School classes attend a ‘Villa Vitality’ day. Half the day is spent with Football Club coaches, exercising and learning football skills, and the other half of the day is an interactive learning session on healthy eating and healthy lifestyles.	Physical Political Sociocultural
Increasing skills of families through activity-based learning	Cooking courses for family members	To increase healthy cooking skills and confidence of family members, and influence the family's nutritional intake	Five week courses on healthy cooking are delivered through schools to parents or other family members, some courses include children. Healthy recipes are distributed to support the course content.	Physical Sociocultural
Information on local leisure opportunities and week-end “taster” sessions for families	To equip parents and families with the knowledge and skills to undertake physical activities with their children in their leisure time	Parents are given information on local sporting and leisure venues and events. They are invited through schools to bring their children to different physical activity taster sessions run on Saturdays. Activities range from cricket and football, to archery, climbing and dry-slope skiing. There is no cost for the activities and transport is provided.	Physical Sociocultural (economic)
Training walk leaders to initiate community walking programmes	To increase walking by families and other community members through organised leisure walks lead by a community member	Community volunteers are recruited through schools to become trained walk leaders. Training is provided to equip volunteers to organise and lead walks in their local community.	Physical Sociocultural

a As defined by the Analysis Grid for Environments Linked to Obesity (Swinburn et al., 1999).
